# Tumor stroma with senescence-associated secretory phenotype in steatohepatitic hepatocellular carcinoma

**DOI:** 10.1371/journal.pone.0171922

**Published:** 2017-03-08

**Authors:** Jee San Lee, Jeong Eun Yoo, Haeryoung Kim, Hyungjin Rhee, Myoung Ju Koh, Ji Hae Nahm, Jin Sub Choi, Kee-Ho Lee, Young Nyun Park

**Affiliations:** 1 Department of Pathology, Yonsei University College of Medicine, Seoul, Republic of Korea; 2 BK21 PLUS Project for Medical Science, Yonsei University College of Medicine, Seoul, Republic of Korea; 3 Integrated Genomic Research Center for Metabolic Regulation, Yonsei University College of Medicine, Seoul, Republic of Korea; 4 Department of Pathology, Seoul National University Bundang Hospital, Seoul National University College of Medicine, Seongnam, Republic of Korea; 5 Department of Surgery, Yonsei University College of Medicine, Seoul, Republic of Korea; 6 Division of Radiation Cancer Research, Korea Institute of Radiological and Medical Science, Seoul, Republic of Korea; 7 Severance Biomedical Science Institute, Yonsei University College of Medicine, Seoul, Republic of Korea; Hunter College, UNITED STATES

## Abstract

Senescence secretome was recently reported to promote liver cancer in an obese mouse model. Steatohepatitic hepatocellular carcinoma (SH-HCC), a new variant of HCC, has been found in metabolic syndrome patients, and pericellular fibrosis, a characteristic feature of SH-HCC, suggests that alteration of the tumor stroma might play an important role in SH-HCC development. Clinicopathological characteristics and tumor stroma showing senescence and senescence-associated secretory phenotype (SASP) were investigated in 21 SH-HCCs and 34 conventional HCCs (C-HCCs). The expression of α-smooth muscle actin (α-SMA), p21^Waf1/Cif1^, γ-H2AX, and IL-6 was investigated by immunohistochemistry or immunofluorescence. SH-HCCs were associated with older age, higher body mass index, and a higher incidence of metabolic syndrome, compared to C-HCC (*P* <0.05, all). The numbers of α-SMA-positive cancer-associated fibroblasts (CAFs) (*P* = 0.049) and α-SMA-positive CAFs co-expressing p21^Waf1/Cif1^ (*P* = 0.038), γ-H2AX (*P* = 0.065), and IL-6 (*P* = 0.048) were greater for SH-HCCs than C-HCCs. Additionally, non-tumoral liver from SH-HCCs showed a higher incidence of non-alcoholic fatty liver disease and a higher number of α-SMA-positive stellate cells expressing γ-H2AX and p21^Waf1/Cif1^ than that from C-HCCs (*P* <0.05, all). In conclusion, SH-HCCs are considered to occur more frequently in metabolic syndrome patients. Therein, senescent and damaged CAFs, as well as non-tumoral stellate cells, expressing SASP including IL-6 may contribute to the development of SH-HCC.

## Introduction

As in Western countries, the prevalence of metabolic syndrome is rapidly increasing in Asia, including Korea [1, 2]. Metabolic syndrome induces non-alcoholic fatty liver disease (NAFLD), which encompasses a broad spectrum of conditions, ranging from simple steatosis to non-alcoholic steatohepatitis, and ultimately cirrhosis [3] Metabolic syndrome patients are reportedly at twice as high a risk for hepatocellular carcinoma (HCC) than normal individuals [4]. Moreover, diabetes and obesity, two major components of metabolic syndrome, increase the risk of HCC in chronic B or C viral patients by approximately 100-fold [5]. Recently, a histologically distinct subtype of HCC showing features of steatohepatitis within tumor regions has been pathologically characterized and introduced as a new HCC category, termed steatohepatitic HCC (SH-HCC) [6–8]. The SH-HCC variant, which is characterized by large droplet steatosis, pericellular fibrosis, inflammation, ballooning, and Mallory-Denk body formation, has been reported to be associated with metabolic syndrome [6–8].

The biological behavior of cancers is influenced not only by neoplastic epithelial cells but also by tumor stromal cells [9]. Cancer-associated fibroblasts (CAFs) (also known as myofibroblasts), a component of the tumoral stroma, have been reported to promote tumor growth, invasion, and angiogenesis. Aggressive biologic behavior and dismal prognosis have also been demonstrated various cancers with abundant CAFs, including HCCs [10–12]. One distinctive pathologic feature of SH-HCC is pericellular fibrosis, compared to conventional HCCs (C-HCCs), which usually show little or no stromal fibrosis. Accordingly, the moleculo-pathological characteristics of the tumor stroma in SH-HCCs might be different from that in C-HCCs.

Cellular senescence encompasses a complex biological process of tumor progression, tumor suppression, aging, and tissue repair. Senescent cells develop a senescence-associated secretory phenotype (SASP) that can affect the behavior of neighboring cells [13]. Reportedly, dietary- and genetically-induced obese mice show enhanced liver inflammation and tumorigenesis promoted by IL-6 [14]. More recently, SASP in hepatic stellate cells (HSCs), along with secretion of various inflammatory and tumor-promoting factors, was found to contribute to HCC development in obese mice [15].

In the present study, we aimed to investigate alterations of the tumor stroma in SH-HCCs, comparing the expression of CAFs, HSCs, senescence-associated proteins, and SASP factors (p21^Waf1/Cif1^, γ-H2AX, and IL-6) between SH-HCCs and C-HCCs.

## Materials and methods

### Case selection and clinicopathologic evaluation

We reviewed the pathological and clinical records of consecutive HCC patients who underwent partial hepatectomy or liver transplantation between 2009 and 2014 from the archives of the Department of Pathology, Yonsei University College of Medicine. This study was approved by the Institutional Review Board of Severance Hospital, Yonsei University College of Medicine, and the need for patient consent was waived (4-2012-0649). Patients who underwent pre-operative chemotherapy or locoregional therapy (such as transarterial chemoembolization or radioactive frequency ablation) were excluded. We also excluded patients with a history of excessive alcohol consumption (defined as >40 g/day). Representative formalin-fixed, paraffin-embedded (FFPE) tissue sections stained with hematoxylin-eosin and Masson’s trichrome were reviewed for all cases.

**Fig 1:**
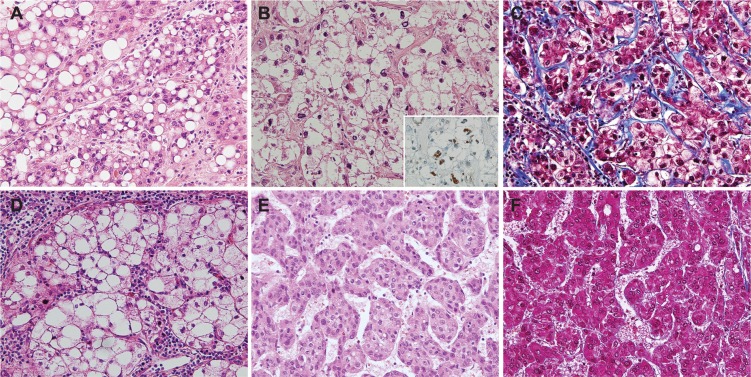
Pathological features of the steatohepatitic hepatocellular carcinoma (SH-HCC) and conventional HCC (C-HCC). A-D) Representative images of SH-HCC showing (A) large-droplet steatosis, (B) ballooning change with Mallory-Denk bodies (inset: Mallory-Denk bodies demonstrated by immunohistochemical stain for ubiquitin), (C) pericellular fibrosis in a chicken-wire pattern, and (D) lymphocytic infiltration. E-F) Representative images of C-HCC without steatosis or fibrosis (A, B, D, E, H-E; C, F, Masson’s trichrome, original magnification x200; inset (B), immunohistochemical stain for ubiquitin, original magnification x400).

The SH-HCCs included in this study showed at least four of the following features in ≥50% of the tumor area: 1) large-droplet fat within the tumor; 2) ballooning change; 3) Mallory-Denk bodies; 4) pericellular fibrosis with a “chicken-wire” appearance; and 5) inflammation, including infiltration of neutrophils and lymphocytes (Fig 1A–1D). The presence or absence of Mallory-Denk bodies was evaluated by immunoreactivity for ubiquitin. For comparison, C-HCCs with the typical histopathological features of HCC were selected (Fig 1E and 1F). Other histopathological features including size, grades of differentiation, and presence of microvascular invasion were evaluated in each HCC. Matching non-neoplastic liver tissue from each case was examined for the presence of NAFLD or chronic hepatitis [16].

Medical records were reviewed to check for the presence of the following metabolic syndrome risk factors: central obesity (waist circumference >90 cm in men and >80 cm in women), hypertriglyceridemia (serum triglycerides ≥150 mg/dLor current use of antidyslipidemic medication), low high-density lipoprotein cholesterol (<40 mg/dL in men and <50 mg/dL in women), diabetes (elevated fasting plasma glucose levels ≥100 mg/dL or current use of anti-diabetic medication), and hypertension (systolic blood pressure ≥130 mmHg or diastolic blood pressure ≥85 mmHg or current use of blood pressure medication). According to the US National Cholesterol Education Program Adult Treatment Panel III (NCEP ATP III, 2001) and International Diabetes Federation criteria, metabolic syndrome was defined by at least two of the following: central obesity, low high-density lipoprotein cholesterol, diabetes, hypertension, and hypertriglyceridemia [17, 18]. Serum hepatitis B virus (HBV) surface antigen (HBsAg) status, anti-hepatitis C virus (HCV), and body mass index (BMI) were also reviewed.

### Immunohistochemistry and immunofluorescence

Immunohistochemistry and immunofluorescence for α-smooth muscle actin (SMA), p21^Waf1/Cip1^, γ-H2AX, IL-6, Ki-67, and ubiquitin were performed using representative sections of FFPE. The complete details of the primary antibodies used are presented in Table 1. Immunohistochemistry was performed using an Envision kit (Dako, Glostrup, Denmark) according to the manufacturer’s instructions. For double immunohistochemistry, the first primary antibody was detected using a Vector Blue Alkaline Phosphatase Substrate Kit III (SK-5300; Vector Laboratories, Burlingame, CA), while the second primary antibody was detected using Dako Envision kit (Dako) and then developed with 3,3-diaminobenzidine. For double immunofluorescence, Alexa fluor 594 (red) goat anti-rabbit IgG and Alexa fluor 488 (green) donkey anti-mouse IgG conjugated antibodies (Invitrogen, Carlsbad, CA) were used. Nuclei were stained with 4’-6’ diamidino-2-phenylindole (DAPI) (Molecular probe, Gaithersburg, MD).

**Table 1:** List of antibodies used for immunohistochemistry and immunofluorescence.

Antibody	Source	Dilution	Antigen retrieval
α-SMA (mouse mAb; 1A4)	Dako (Glostrup, Denmark)	1:1000	Microwave, citrate (pH 6.0)
α-SMA (rabbit pAb)	Abcam (Cambridge,UK)	1:300	Microwave, citrate (pH 6.0)
p21^Waf1/Cip1^ (rabbit mAb; 12D1)	Cell signaling (Danvers, MA)	1:50	Microwave, citrate (pH 6.0)
γ-H2AX (rabbit mAb; 20E3)	Cell signaling (Danvers, MA)	1:150	Microwave, citrate (pH 6.0)
IL-6 (rabbit pAb)	Abcam (Cambridge, UK)	1:100	Protease K
Ki-67 (mouse mAb; MIB-1)	Dako (Glostrup, Denmark)	1:100	Microwave, citrate (pH 6.0)
Ubiquitin (rabbit pAb)	Dako (Glostrup, Denmark)	1:200	Automated immunostainer

Abbreviations: α-SMA, α-smooth muscle actin; mAb, monoclonal antibody; pAb, polyclonal antibody.

The number of α-SMA-positive CAFs or α-SMA-positive HSCs was counted in 20 randomly selected, high-power fields (x400 magnification). The percentage of CAFs or HSCs co-expressing p21^Waf1/Cip1^ and α-SMA was calculated by dividing the total number of p21^Waf1/Cip1^ and α-SMA co-stained cells by the total number of α-SMA-positive cells and multiplying by 100%. The percentage of CAFs or HSCs co-expressing γ-H2AX/α-SMA, IL-6/α-SMA and Ki-67/α-SMA were evaluated similarly. For IL-6, the staining intensity was graded on a scale of 0–3 (0, negative; 1, weakly positive; 2, moderately positive; and 3, strongly positive), and the extent of distribution was rated on a scale of 0–4 (0, expression in <5% of cells; 1, 5–25%; 2, 26–50%; 3, 51–75%; and 4, 76–100%). Histoscores were calculated as the sum of the intensity and distribution scores. Positive expression was defined as a histoscore of 4–7; a score of 0–3 was regarded as negative.

For tumoral and non-tumoral hepatocytes, the histoscores for p21^Waf1/Cip1^, *γ*-H2AX, and Ki-67 labeling indices (LIs) were calculated as the percentage of positively stained nuclei, and at least 1000 cells were counted in random areas of tissue sections.

### DNA extraction and HBV DNA nested PCR

To detect occult HBV infection, the liver tissues of 20 patients who were negative for serum HBsAg were analyzed by HBV DNA-nested PCR. Total DNA was extracted from 15 snap frozen liver tissues using a Qiagen QIAamp DNA Mini Kit (Qiagen, Hilden, Germany) and from five FFPE liver tissues using a ReliaPrep™ FFPE gDNA Miniprep System (Promega, Madison, WI) according to the manufacturers’ instructions. Four different in-house, nested-PCR amplification assays were applied to detect PreS-S, Precore–core, Pol, and X HBV genomic regions of HBV. We considered a case to be positive for HBV DNA when at least two different viral genomic regions were detected [19]. The primer sets and PCR conditions are listed in S1 Table. PCR was performed with the AccuPower PCR Premix (Bioneer, Daejeon, Korea), containing 10 pM of primers and 250 ng of genomic DNA.

### Statistical analyses

Data were analyzed using SPSS software, version 20 (SPSS Inc., Chicago, IL). Differences between groups were analyzed using Student’s t-test, χ^2^-test, and Fisher’s exact test, as deemed appropriate. Univariable survival analyses were performed for overall and disease-free survival using Kaplan-Meier’s method and log-rank tests. Statistical significance was reached when *P* <0.05, and *P* <0.1 was reported as a trend.

## Results

### Clinicopathological characteristics of steatohepatitic HCC

The clinical features of twenty-one cases of SH-HCCs and 34 cases of C-HCCs are summarized in Table 2. Patients with SH-HCC showed significantly older age and higher BMI, compared to C-HCC patients (*P* = 0.003 and *P* = 0.027, respectively). Central obesity, diabetes, and hypertriglyceridemia were more frequently seen in SH-HCC patients than in C-HCC patients (*P* <0.05, all), and metabolic syndrome was more frequently found in SH-HCC patients (n = 15, 71.4%) than in C-HCC patients (n = 14, 41.2%) (*P* = 0.029). Chronic HBV infection was present in 15 (71.4%) SH-HCCs and 29 (85.3%) C-HCCs, including occult HBV infection (4 cases in SH-HCCs and 5 cases in C-HCCs), and there was no significant difference between the two groups. Most patients with metabolic syndrome also showed chronic HBV infection: 73.3% (11/15) of SH-HCCs and 78.6% (11/14) of C-HCCs. Among those with chronic HBV infection, four cases (4/15, 26.7%) of SH-HCCs and 18 cases (18/29, 62.1%) of C-HCCs showed HBV infection only without metabolic syndrome. Anti-HCV was not present in any patient from either group.

**Table 2:** Clinicopathological charaterisitcs of the steatohepatitic and conventional hepatocellular carcinoma patients.

		SH-HCC (n = 21)	C-HCC (n = 34)	P value*
Age (years)^a^		66.7 ± 8.4	58.5 ± 10.1	**0.003**
Sex (male:female)		8:13	18:16	0.284
Body mass index (kg/m^2^) ^a^		26.0 ± 4.6	23.7 ± 2.7	**0.027**
Central obesity		12 (57.1%)	11 (32.4%)	**0.012**
Low HDL cholesterol		5 (23.8%)	5 (14.7%)	0.387
Diabetes		12 (57.1%)	10 (29.4%)	**0.041**
Hypertension		10 (47.6%)	14 (41.2%)	0.640
Hypertriglyceridemia		5 (23.8%)	1 (2.9%)	**0.028**
Metabolic syndrome		15 (71.4%)	14 (41.2%)	**0.029**
Chronic HBV infection		15 (71.4%)	29 (85.3%)	0.300
Serum HBsAg (+)		11 (52.4%)	24 (70.6%)	0.392
Occult HBV infection		4 (19.1%)	5 (14.7%)	0.674
Tumoral pathology				
Tumor size (cm)^a^		3.3 ± 1.5	4.2 ± 3.5	0.308
Differentiation				
	Ⅰ	2 (9.5%)	0 (0.0%)	0.086
	Ⅱ	11 (52.4%)	12 (35.3%)	
	Ⅲ	8 (38.1%)	21 (61.8%)	
	Ⅳ	0 (0.0%)	1 (2.9%)	
Microvessel invasion		8 (38.1%)	15 (44.1%)	0.660
Non-tumor pathology				
NAFLD alone		4 (19.0%)	2 (5.9%)	**0.001**
NAFLD with chronic hepatitis		14 (66.7%)	10 (29.4%)	
Chronic hepatitis alone		3 (14.3%)	22 (64.7%)	

Abbreviations: HDL, high-density lipoprotein; HBV, hepatitis B virus; HBsAg, hepatitis B virus surface antigen; NAFLD, non-alcoholic fatty liver disease.

* Fisher’s exact test, Pearson chi-square and Student’s t-test. Statistically significant *P* values are expressed in bold font.

^a^ Mean ± standard deviation.

The pathological features of the SH-HCCs and C-HCCs are summarized in Table 2. Tumor size, differentiation, and microvascular invasion were not significantly different between the two HCC groups. In the non-neoplastic livers, NAFLD was more frequently found in SH-HCCs than in C-HCCs (*P* = 0.001). NAFLD was noted in 18 (85.7%) SH-HCC patients, including four cases of NAFLD alone and 14 cases of NAFLD with co-existing chronic hepatitis. In contrast, the background liver of C-HCCs showed NAFLD in 12 cases (35.3%), including two cases of NAFLD alone and 10 cases of NAFLD with co-existing chronic hepatitis.

### Expressions of p21^Waf1/Cip1^, γ-H2AX, and IL-6 in tumoral regions of steatohepatitic HCC *vs*. conventional HCC

**Fig 2:**
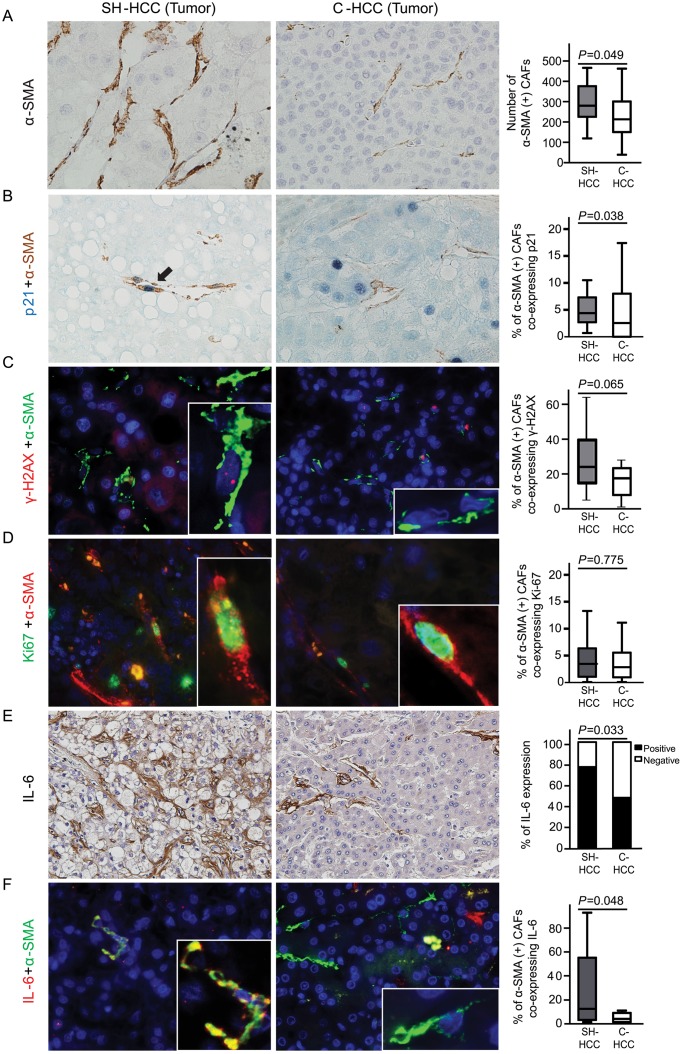
Cancer-associated fibroblasts (CAFs) expressing p21^Waf1/Cip1^, γ-H2AX, and IL-6 in tumoral regions of steatohepatitic hepatocellular carcinomas (SH-HCCs) and conventional HCCs (C-HCCs). A) CAFs expressing α-SMA are more frequently found in SH-HCCs than in C-HCCs. B) Double immunohistochemical stain demonstrates nuclear p21^Waf1/Cip1^ in blue and cytoplasmic α-SMA in brown. CAFs co-expressing p21^Waf1/Cip1^ and α-SMA are more frequently seen in SH-HCCs than in C-HCCs. (C) Double immunofluorescence images of γ-H2AX (red) and α-SMA (green). CAFs co-expressing γ-H2AX and α-SMA are relatively higher in SH-HCCs than in C-HCCs. (D) Double immunofluorescence images of Ki-67 (green) and α-SMA (red). There is no difference between groups. (E) Greater expression of IL-6, detected by immunohistochemistry, in SH-HCCs than in C-HCCs. (F) Double immunofluorescence of IL-6 (red) and α-SMA (green). Nuclei were stained with DAPI. CAFs co-expressing IL-6 and α-SMA are significantly more abundant in SH-HCCs than in C-HCCs. The merged fluorescence images of boxed areas are further magnified in the insets. Box plot graphs in the right column demonstrate comparisons between the two groups (A-D, F, original magnification x400; E, original magnification x200). α-SMA, α-smooth muscle actin.

A significantly greater number of α-SMA-positive CAFs were seen in tumoral regions of SH-HCCs, compared to C-HCCs (mean ± SD: 295.4 ± 100.47 for SH-HCCs and 233.9 ± 111.56 for C-HCCs per 20 high-power fields, *P* = 0.049) (Fig 2A). The expression status of the senescence marker p21^Waf1/Cip1^ and DNA damage marker γ-H2AX was evaluated in CAFs. The percentage of CAFs co-expressing nuclear p21^Waf1/Cip1^ and cytoplasmic α-SMA was significantly higher in SH-HCCs than in C-HCCs (5.9 ± 4.69% *vs*. 4.2 ± 5.36%, *P =* 0.038) (Fig 2B). The percentage of CAFs co-expressing nuclear γ-H2AX and cytoplasmic α-SMA also tended to be higher in SH-HCCs than in C-HCCs (27.3 ± 16.50% *vs*. 19.2 ± 15.43%, *P =* 0.065) (Fig 2C). There were no significant differences in the proliferative activity of CAFs (reflected by the co-expression of nuclear Ki-67 and cytoplasmic α-SMA) between the two groups (4.6 ± 4.89% *vs*. 3.9 ± 4.13%, *P =* 0.775) (Fig 2D). IL-6 expression was mainly found in tumoral stroma, and was more highly expressed in SH-HCCs than in C-HCCs (*P* = 0.033) (Fig 2E). In addition, double immunofluorescence staining for IL-6 and α-SMA revealed co-expression of IL-6/α-SMA in 29.3 ± 33.61% and 7.0 ± 14.10% of CAFs in SH-HCCs and C-HCCs, respectively; this was a statistically significant difference (*P* = 0.048) (Fig 2F). Taken together, these findings indicate that damaged and senescent CAFs expressing IL-6 are more common in SH-HCCs than in C-HCCs.

Additionally, we evaluated expression of p21^Waf1/Cip1^ and γ-H2AX in tumoral hepatocyte-like epithelial cells of SH-HCCs and C-HCCs, and there was no significant difference in p21^Waf1/Cip1^ and γ-H2AX LIs. There was also no significant difference in Ki-67 LIs between the tumoral hepatocyte-like epithelial cells of SH-HCCs and C-HCCs (S1A–S1C Fig).

### Expressions of p21^Waf1/Cip1^, γ-H2AX, and IL-6 in non-tumoral regions of steatohepatitic HCC *vs*. conventional HCC

**Fig 3:**
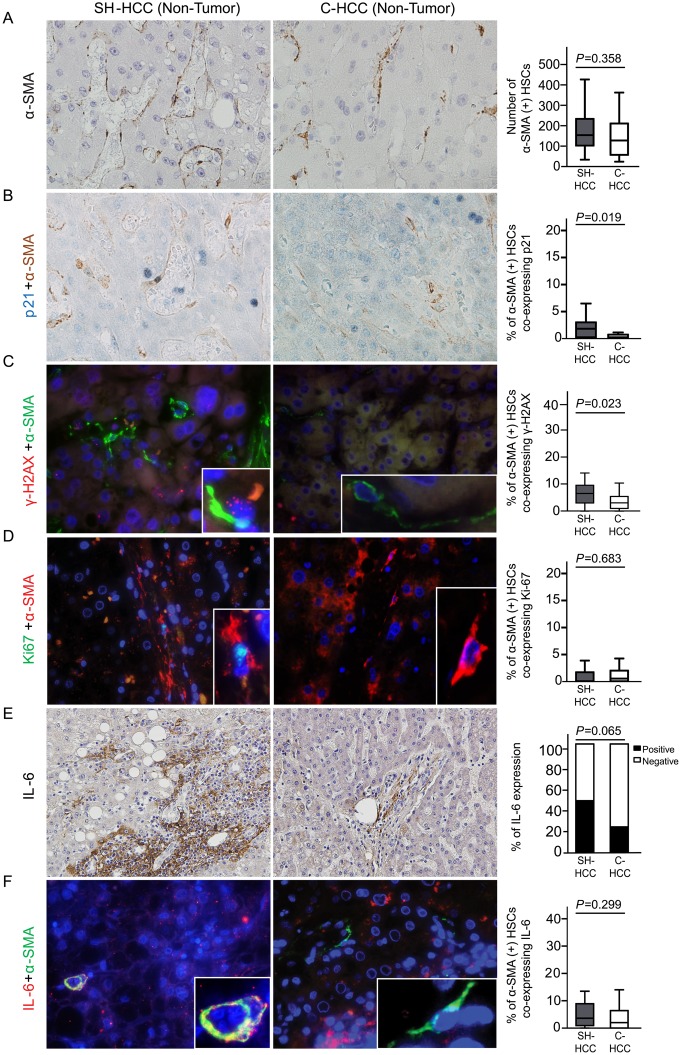
Non-tumoral hepatic stellate cells (HSCs) expressing p21^Waf1/Cip1^, γ-H2AX, and IL-6 in non-tumoral regions of SH-HCCs and C-HCCs. (A) Non-tumoral HSCs expressing α-SMA show no differences in number between the two groups. (B) Double immunohistochemical stain reveals nuclear p21^Waf1/Cip1^ in blue (arrow) and cytoplasmic α-SMA in brown. Non-tumoral HSCs co-expressing p21^Waf1/Cip1^ and α-SMA are more frequently seen in non-tumoral regions of SH-HCCs compared to that for C-HCCs. (C) Double immunofluorescence images of γ-H2AX (red) and α-SMA (green). Non-tumoral HSCs co-expressing γ-H2AX and α-SMA are higher in non-tumoral regions of SH-HCCs, compared to that for C-HCCs. (D) Double immunofluorescence images of Ki-67 (green) and α-SMA (red) showing no difference between groups. (E) IL-6 expression, detected by immunohistochemistry, is relatively higher in the stroma of non-tumoral regions of SH-HCCs, compared to that for C-HCCs. (F) Double immunofluorescence of IL-6 (red) and α-SMA (green) show no difference between groups. Nuclei were stained with DAPI. The merged fluorescence images of boxed areas are further magnified in the insets. Box plot graphs in the right column demonstrate comparisons between the two groups (A-D, F, original magnification x400; E, original magnification x200). α-SMA, α-smooth muscle actin.

In non-tumoral regions, the numbers of α-SMA-expressing non-tumoral HSCs (per 20 high-power fields) were 167.7 ± 95.81 (mean ± SD) and 144.8 ± 125.50 in SH-HCCs and C-HCCs, respectively, and the difference was not statistically significant (*P* = 0.358) (Fig 3A). The percentage of non-tumoral HSCs co-expressing nuclear p21^Waf1/Cip1^ and cytoplasmic α-SMA was significantly higher in non-tumoral regions of SH-HCCs than those of C-HCCs (2.0 ± 2.42% *vs*. 0.7 ± 1.44%, *P =* 0.019) (Fig 3B). The percentage of HSCs co-expressing γ-H2AX and α-SMA was also higher in non-tumor regions of SH-HCCs than those of C-HCCs (7.6 ±7.01% *vs*. 3.8 ± 3.16%, *P =* 0.023) (Fig 3C). Co-expression of Ki-67 and α-SMA was very rarely found in non-tumoral HSCs, without significant differences between the two groups (0.8 ± 1.24% *vs*. 1.1 ± 1.28%, *P =* 0.683) (Fig 3D). The expression of IL-6 was mainly found in the stroma of portal tracts and fibrous septa of non-tumoral regions, and although not statically significant, was relatively highly expressed in SH-HCCs than in C-HCCs (*P =* 0.065) (Fig 3E). The percentage of non-tumoral HSCs that co-expressed IL-6 and α-SMA was very low, and showed no significant difference between the two HCC groups (5.4 ± 5.90% *vs*. 4.0 ± 5.34%, *P* = 0.299) (Fig 3F).

Non-tumoral hepatocytes were also evaluated for p21^Waf1/Cip1^, γ-H2AX, and Ki-67 LIs, and no significant differences were seen in the expression of these markers between SH-HCCs and C-HCCs (*P >*0.05 for all) (S1D–S1F Fig).

### Survival analysis of steatohepatitic and conventional HCCs

The median follow-up time after surgical resection was 30.5 months (range, 1–73), and one patient with C-HCC who underwent liver transplantation was excluded from the survival analysis. Kaplan-Meier plots revealed no significant differences between SH-HCCs (n = 21) and C-HCCs (n = 33) in both disease-free (*P* = 0.602) and overall survival (*P* = 0.709) (S2 Fig).

## Discussion

Recently, a histologically distinct subtype of HCC, termed SH-HCC has been introduced and one of distinctive pathologic features of SH-HCC is pericellular fibrosis. In this study, α-SMA-positive CAFs, which are considered to contribute to pericellular fibrosis, were more frequent in the tumoral regions of SH-HCCs than those for C-HCCs. In addition, we found that markers of cellular senescence, p21^Waf1/Cip1^, and DNA damage, γ-H2AX, are more highly expressed in CAFs from SH-HCCs than those from C-HCCs. In contrast, the proliferative activity of CAFs showed no significant difference between two groups. Thus, our results suggested that senescent and damaged CAFs might be important in the pathogenesis of SH-HCCs. Cellular senescence was previously thought to be a barrier to tumorigenesis; however, recently, it has also been reported to promote carcinogenesis. The DNA damage signaling pathway leads to the activation of p53 tumor suppressor, which in turn may cause transient arrest of the cell cycle in addition to DNA repair, and ultimately leading to cancer suppression [20]. In contrast, loss of p53 activity in senescent or damaged fibroblasts enhances SASP, which can drive cancer and aging [13]. An altered tissue microenvironment induced by senescent cells has been proposed to contribute to increased cancer occurrence in old aged populations, and senescent human fibroblasts were reported to promote proliferation and tumorigenesis of mutant epithelial cells in an *in vitro* study [21]. Indeed, the patients with SH-HCCs were older than those with C-HCCs in this study.

IL-6, one of SASP factors, is a pro-inflammatory signaling protein that encourages tumor growth, and exerts its oncogenic activity by triggering downstream STAT-3 and ERK pathways [14, 22]. In our study, IL-6 was mainly expressed in CAFs, and was more highly expressed in SH-HCCs than in C-HCCs, suggesting that IL-6, induced by a senescent phenotype in CAFs, may alter the tumor stroma, which is important in the development of SH-HCCs. IL-6 is also known to be associated with metabolic disorders, and has been found to be up-regulated in NAFLD and obesity-related HCC [14, 23].

In addition, we found NAFLD more often in the background liver of SH-HCC patients than those from C-HCC patients. In non-neoplastic liver, HSCs undergo phenotypic conversion from quiescent retinoid-storing cells to active myofibroblasts in response to stimuli, including fatty change, reactive oxygen species generation, and DNA damage, and ultimately affect fibrosis progression. In chronic liver disease, including NAFLD and chronic viral hepatitis, increased cytokine production from HSCs and immune cells has been reported to promote hepatocarcinogenesis [11]. Interestingly, the expression of p21^Waf1/Cip1^ and γ-H2AX in non-tumoral HSCs was significantly greater in SH-HCCs than in C-HCCs. Moreover, expression of IL-6 was relatively higher in the background liver of SH-HCCs, compared to C-HCCs. These findings suggest that the development of SH-HCC is also influenced by SASP of senescent and damaged HSCs in the background liver with NAFLD.

Recently, several changes in the composition of the intestinal microbiomes of dietary- and genetically-mutated obese mice have been demonstrated, and the changes have been shown to lead to the production of deoxycholic acid, a secondary bile acid known to cause DNA damage. This, in turn, provoked HSCs to undergo senescence and to produce SASP factors, such as IL-6, ultimately leading to the development of HCC [15]. Our data of human SH-HCCs support this study. Therefore, senescent CAFs and HSCs with SASP, which are characteristic of tumoral and non-tumoral stroma of SH-HCCs, are considered to be important in the development of SH-HCCs, and they might be promoted by gut microbial metabolites in patients with metabolic syndrome. Further study thereon is needed.

Previous studies have shown SH-HCC to be associated with metabolic syndrome [6–8], and this study also revealed an association between SH-HCCs, higher body mass index, and a higher incidence of metabolic syndrome, compared to C-HCC. SH-HCC has also been reported in chronic C viral hepatitis patients with or without metabolic syndrome; however, the association of SH-HCC with HBV, which is the main etiology of HCC in Asia, including Korea, remains unclear [24]. The natural history of chronic HBV infection ranges from the replicative phase with active liver disease (hepatitis B e antigen [HBeAg]-positive hepatitis) to low or non-replicative phase with HBeAg seroconversion and remission of liver disease (inactive carriers). Subsequently in some cases, spontaneous hepatitis B surface antigen (HBsAg) seroclearance, which is regarded as a surrogate marker of resolved hepatitis B, may occur with an estimated annual incidence of 0.1–2% with geographic variations. In the patients with occult HBV infection after seroclearance of circulating HBsAg, HBV DNA is persistently detected in the liver tissues, and the risk of HCC remains although necroinflammation is markedly improved. Previously, our group reported that 5 of 49 (10.2%) patients with occult HBV infection were noted to have HCC during a mean follow-up period of 19.6 months after HBsAg seroclearance [25]. In this study, to thoroughly investigate the association between SH-HCCs and HBV, we checked the serum HBsAg by reviewing medical record, and for the patients with negative serum HBsAg, occult HBV infection was examined like followings: total DNA was extracted from the liver tissues and four different nested-PCR amplification assays were applied to detect PreS-S, Precore–core, Pol, and X HBV genomic regions of HBV. We considered to be positive for HBV DNA when at least two different viral genomic regions were detected. In this study, the incidence of chronic HBV infection showed no significance difference between SH-HCCs and C-HCCs, although the incidence of metabolic syndrome was higher in SH-HCCs compared to C-HCCs. Actually, the majority of SH-HCCs (15/21, 71.4%) and C-HCCs (29/34, 85.3%) showed chronic HBV infection. Among them, four SH-HCCs (4/15, 26.7%) and five C-HCCs (5/29, 17.2%) demonstrated occult HBV infection. In non-neoplastic liver with occult HBV infection, all of four SH-HCCs showed NAFLD with chronic hepatitis, and C-HCCs revealed two cases of NAFLD and three cases of NAFLD with chronic hepatitis, where the necroinflammatory activity was low. Interestingly, four cases of SH-HCC in this study showed HBV infection only without metabolic syndrome. Previous studies on transgenic mice have shown that HBV protein X (HBx) can up-regulate lipogenic genes and promote steatosis [26, 27]. Moreover, in HBx transgenic mice fed a high fat diet, fatty acid was found to stabilize HBx protein and thereby promote steatohepatitis [28]. Therefore, HBV itself might be involved in the lipogenesis of HCC, one of the main features of SH-HCC.

In conclusion, our results suggest that SH-HCC is a distinctive variant of HCC, which develops more frequently in metabolic syndrome patients, and that senescent and damaged CAFs, as well as non-tumoral stellate cells with SASP, including IL-6 expression, may contribute to the development of SH-HCC.

## Supporting information

S1 Fig **Stack graph and box plots show p21**^**Waf1/Cip1**^ **expression, labelling indices of γ-H2AX and Ki-67 in tumoral hepatocyte-like cells (A-C) and non-tumoral hepatocytes (D-F) of steatohepatitic and conventional HCCs.** SH-HCC, steatohepatitic HCC; C-HCC, conventional HCC.
(EPS)

S2 Fig **Kaplan–Meier’s plot analysis for (A) disease-free and (B) overall survival in steatohepatitic and conventional HCC patients.** SH-HCC, steatohepatitic HCC; C-HCC, conventional HCC.
(EPS)

S1 Table Sequences of the primers used for the HBV DNA nested PCR experiments. (DOCX)

S1 Data Supporting data. (XLSX)
