# Supplementary material for: Tumor stroma with senescence-associated secretory phenotype in steatohepatitic hepatocellular carcinoma
Source: PLoS One. 2017 Mar 8;12(3):e0171922. doi: 10.1371/journal.pone.0171922 (PMC5342190; doi:10.1371/journal.pone.0171922)
Supplement: S1 Table — (DOCX) [file pone.0171922.s003.docx]

**S1 Table.** Sequences of the primers used for the HBV DNA nested PCR experiments

| Primer set | Sense primers | Antisense primers | T_a_^b^ (°C ) |
| --- | --- | --- | --- |
|  |  |  |  |
| PreS-S | 5′-GGTCACCATATTCTTGGGAA-3′ | 5′-AATGGCACTAGTAAACTGAG-3′ | 47.4 |
| PreS-S^a^ | 5′-AATCCAGATTGGGACTTCAA-3′ | 5′-CCTTGATAGTCCAGAAGAAC-3′ | 47.4 |
| Precore-core | 5′-GCCTTAGAGTCTCCTGAGCA-3′ | 5′-GTCCAAGGAATACTAAC-3′ | 47.8 |
| Precore-core^a^ | 5′-CCTCACCATACTGCACTCA-3′ | 5′-GAGGGAGTTCTTCTTCTAGG-3′ | 50.2 |
| Pol | 5′-CGTCGCAGAAGATCTCAATC-3′ | 5′-CCTGATGTGATGTTCTCCATG-3′ | 50.7 |
| Pol^a^ | 5′-CCTTGGACTCATAAGGT-3′ | 5′-TTGAAGTCCCAATCTGGATT-3 | 45.7 |
| X | 5′-CCATACTGCGGAACTCCTAGC-3′ | 5′-CGTTCACGGTGGTCTCCAT-3′ | 57.4 |
| X^a^ | 5′-GCTAGGCTGTGCTGCCAACTG-3′ | 5′-CGTAAAGAGAGGTGCGCCCCG-3′ | 59.7 |

^a^Applied in the second round; ^b^Anealing temperature
